# High sound pressure piezoelectric micromachined ultrasonic transducers using sputtered potassium sodium niobate

**DOI:** 10.1038/s41378-024-00841-y

**Published:** 2024-12-27

**Authors:** Fan Xia, Yande Peng, Wei Yue, Mingze Luo, Megan Teng, Chun-Ming Chen, Sedat Pala, Xiaoyang Yu, Yuanzheng Ma, Megha Acharya, Ryuichi Arakawa, Lane W. Martin, Liwei Lin

**Affiliations:** 1 Department of Mechanical Engineering, University of California, Berkeley, CA 94720 USA; 2 School of Electronic Science and Engineering, Southeast University, Nanjing, Jiangsu 210096 China; 3 Bioengineering and Biomedical Engineering, Xi’an Jiaotong University, Xi’an, Shaanxi 710049 China; 4 Tsinghua Shenzhen International Graduate School, Tsinghua University, Shenzhen, 518055 China; 5 Department of Materials Science and Engineering, University of California, Berkeley, CA 94720 USA; 6 Scientific Research Laboratory Div., Niterra Co., Ltd, Nagoya, 461-0005 Japan; 7 Departments of Materials Science and NanoEngineering, Chemistry, and Physics and Astronomy and the Rice Advanced Materials Institute, Rice University, Houston, TX 77005 USA

**Keywords:** Engineering, Nanoscience and technology

## Abstract

This work presents air-coupled piezoelectric micromachined ultrasonic transducers (pMUTs) with high sound pressure level (SPL) under low-driving voltages by utilizing sputtered potassium sodium niobate K_0.34_Na_0.66_NbO_3_ (KNN) films. A prototype single KNN pMUT has been tested to show a resonant frequency at 106.3 kHz under 4 V_p-p_ with outstanding characteristics: (1) a large vibration amplitude of 3.74 μm/V, and (2) a high acoustic root mean square (RMS) sound pressure level of 105.5 dB/V at 10 cm, which is 5–10 times higher than those of AlN-based pMUTs at a similar frequency. There are various potential sensing and actuating applications, such as fingerprint sensing, touch point, and gesture recognition. In this work, we present demonstrations in three fields: haptics, loudspeakers, and rangefinders. For haptics, an array of 15 × 15 KNN pMUTs is used as a non-contact actuator to provide a focal pressure of around 160.3 dB RMS SPL at a distance of 15 mm. This represents the highest output pressure achieved by an airborne pMUT for haptic sensation on human palms. When used as a loudspeaker, a single pMUT element with a resonant frequency close to the audible range at 22.8 kHz is characterized. It is shown to be able to generate a uniform acoustic output with an amplitude modulation scheme. In the rangefinder application, pulse-echo measurements using a single pMUT element demonstrate good transceiving results, capable of detecting objects up to 2.82 m away. As such, this new class of high-SPL and low-driving-voltage pMUTs could be further extended to other applications requiring high acoustic pressure and a small form factor.

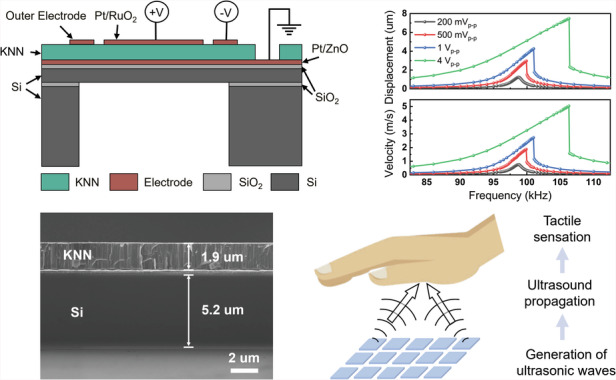

## Introduction

Ultrasonic transducers have been widely used in object detection^1^, non-destructive testing (NDT)^2,3^, biomedical imaging^4,5^, and therapeutic treatments^6,7^. Compared with bulk ultrasonic transducers, piezoelectric micromachined ultrasonic transducers (pMUTs) with small footprints offer advantages of low power consumption and wide bandwidth for applications in consumer electronics and the Internet of Things (IoT), such as range-finding^8,9^, gesture recognition^10,11^, fingerprint sensing^12,13^, and 3D imaging^14,15^. However, these small sensors have relatively low output pressure, which limits signal transmission in various applications. For instance, the state-of-the-art AlN-based pMUT array has only achieved a travel distance of 4 m^16^. To extend pMUTs’ use in applications, such as mid-air haptics^17–19^, loudspeakers^20,21^, and acoustic tweezers^22^, the main challenge lies in achieving a high output sound pressure level (SPL).

The transmitting characteristics of pMUTs are primarily defined by the mechanical structural design and the active piezoelectric material, and the quest for better performance leads to a need for new materials. Extensive efforts have been made in optimizing the device structures, such as new design configurations^23,24^, optimal diaphragm geometries^25–29^, and modified boundary conditions^30–32^. For instance, F. Sammoura et al.^23^ demonstrated a two-port pMUT with the differential driving scheme to double the acoustic power output per input voltage. S. Akhbari et al.^24^ constructed the dual-electrode bimorph (DEB) architecture to realize a vibration displacement four times that of traditional single-electrode unimorph pMUTs. The mode shape of the diaphragm also affects the performance: flexurally-suspended membrane^25^ and holes^26,27^ could transform the mode shape from fully clamped to piston-like, which improves the transmission. Besides, experimental and theoretical work also shows that applying pinned-^31^ or free-^32^ boundary conditions can be beneficial. Aside from the structural design, the active piezoelectric material also plays an important role in the performance, which could be characterized by the piezoelectric coefficient ${e}_{31,f}$. The most studied piezoelectric material, AlN, has a low ${e}_{31,f}$ of −1 C/m^2^. The piezoelectric coefficient can be increased by tuning the material compositions^33–35^. For example, 36% scandium-substituted AlN (ScAlN) films^33^ have an improved ${e}_{31,f}$ of around −2.3 C/m^2^. Other materials, like lead zirconate titanate (PbZr_1-x_Ti_x_O_3_ or PZT)^36,37^, can produce relatively high output pressure, but the presence of lead and their low receiving sensitivity due to high dielectric constant raise concerns for use in real applications. As such, there is a need for new piezoelectric materials to further improve the pMUT performance.

Lead-free piezoelectric materials such as barium titanate (BaTiO_3_)^38^, lithium niobate (LiNbO_3_)^39,40^ and potassium sodium niobate (K,Na)NbO_3_^41–43^ have sparked extensive interest. For example, sol-gel-based KNN pMUT^44^ has exhibited a 1.25 μm/V vibration amplitude under the resonance at 66.2 kHz. Meanwhile, sputtered KNN films^43^ can be batch fabricated as the active layers in pMUTs^45^. Here, we report air-coupled pMUTs based on sputtered, lead-free K_0.34_Na_0.66_NbO_3_ film with a high piezoelectric coefficient (${e}_{31,f}$ ≈ −8 to −10 C/m^2^) and a relatively low dielectric constant (${\varepsilon }_{r}$ ≈260–300). Results show an excellent transmission sensitivity as high as 105.5 dB/V at 10 cm away, which is 5–10 times higher than those of AlN-based pMUTs at a similar frequency. Potential applications in haptics, loudspeakers, and rangefinders have been demonstrated to highlight the advantages of such high-SPL KNN pMUTs. Specifically, (1) an array of 15 × 15 KNN pMUT elements is used to generate haptic stimulation at a frequency within human perception range through pulse-width modulation; (2) a single pMUT with a resonant frequency close to the audible range is tested as a loudspeaker by using the amplitude modulation (AM) scheme; and (3) good transceiving ability is demonstrated by using a single pMUT as an airborne rangefinder based on pulse-echo measurements.

## Results and discussion

### Design

**Fig. 1:**
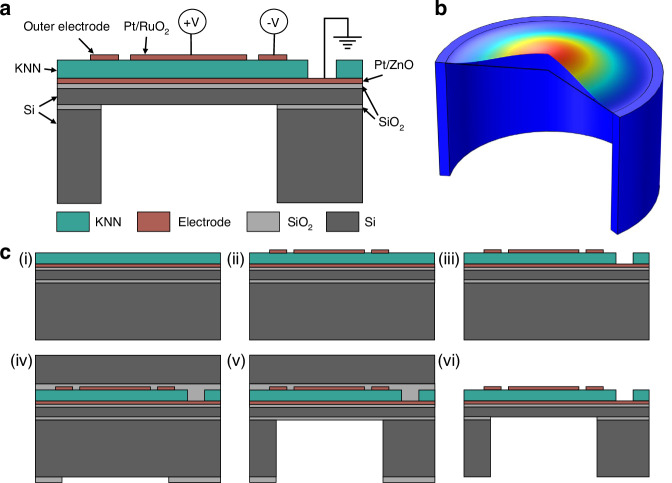
Structure and fabrication process. **a** Schematic cross-section of a circular-shape, unimorph KNN pMUT with the dual-electrode design. **b** Simulation result of the fundamental flexural mode shape. **c** Fabrication process: (i) bottom electrode and KNN film deposition on the SOI wafer; (ii) top electrode deposition and patterning; (iii) KNN film etching for via openings; (iv) oxide hard mask formation and handle wafer bonding; (v) backside Si etching with buried oxide as the etching stop; (vi) removal of the handle wafer and oxide layers

There are three energy domains in a pMUT’s transduction process: electrical, mechanical, and acoustic. PMUTs utilize electric-mechanical-acoustic coupling to convert electrical excitation signals into acoustic waves. As shown in Fig. 1a, the working diaphragm typically comprises a piezoelectric layer and an elastic layer, which is known as a unimorph diaphragm. When an external electric field is applied across the diaphragm, the piezoelectric layer generates in-plane strain through the converse piezoelectric effect. The elastic layer shifts the neutral plane of the entire layer stack away from the midplane of the piezoelectric layer, generating a bending moment that creates out-of-plane displacement^46,47^. When an alternating current (AC) voltage is applied, the diaphragm vibrates periodically in the transverse direction, which is known as the flexural mode. This mechanical vibration pushes the surrounding medium particles to vibrate and ultimately emits an acoustic wave into the environment. The electromechanical coupling coefficient, which indicates the proportion of the total input electrical energy stored as mechanical energy^47,48^, is highly dependent on the piezoelectric coefficient ${e}_{31,f}$. A higher ${e}_{31,f}$ results in a greater electromechanical coupling coefficient, inducing more energy to be converted into mechanical deformation. Therefore, the high ${e}_{31,f}$ value around −8 to −10 C/m^2^ from the sputtered KNN film is beneficial for improving the transmission performance.

Here, the KNN pMUT is designed with a circular unimorph diaphragm consisting of a 2-μm-thick sensor-type KNN film^43,45^ as the active piezoelectric layer and a 5-μm-thick silicon device layer as the elastic layer. The dual-electrode geometry features a circular-shaped inner electrode with a radius equal to 67% of the diaphragm radius and a ring-shaped outer electrode. A differential drive is applied by exciting the inner and outer electrodes with opposite polarity to enhance the vibration displacement and output pressure. The simulated fundamental flexural operation mode with the clamped boundary condition is shown in Fig. 1b. The inner and outer portions experience stress of opposite polarity (tensile or compressive) with the inflection point located at ~67% of the radius^47^. The differential drive configuration maximizes the use of the entire piezoelectric diaphragm for increased outputs.

### Fabrication process

The detailed fabrication process is shown in Fig. 1c. The KNN film deposition process has been previously reported^43,45^. The pMUT fabrication starts with the deposition of a 25-nm-thick zinc oxide (ZnO) adhesion layer and a 200-nm-thick platinum (Pt) bottom electrode layer on a 6-inch silicon-on-insulator (SOI) wafer, which consists of a 5-μm-thick silicon (Si) device layer with a 200-nm-thick thermal oxide layer, 1-μm-thick buried silicon oxide (BOX) layer and a 610-μm-thick (100) Si handle substrate. A 2-μm-thick KNN film, serving as the active piezoelectric layer, is then deposited via a radio frequency (RF) magnetron-sputtering process at 500 °C on top of the Pt/ZnO bottom electrode layer. A stoichiometric (K_0.32_ Na_0.68_)NbO_3_ ceramic material with less than 0.4 at.% of Cu and/or Mn is used as the sputtering target^43^. Subsequently, a 10-nm-thick ruthenium oxide (RuO_2_) layer and a 100-nm-thick Pt layer are sputtered and patterned as the inner circular and outer ring top electrodes, where RuO_2_ is used to promote the adhesion strength between the Pt and KNN film. The opening vias to access the bottom electrode are created by patterning the KNN film through a wet-etching process, using an etchant solution consisting of hydrogen peroxide (H_2_O_2_), ammonium hydroxide (NH_4_OH), and hydroxyethylidene diphosphonic acid (HEDP) in a 75 °C water bath. Finally, the backside silicon cavity is defined by a silicon deep reactive-ion etching (DRIE) process. Before the DRIE process, a layer of silicon oxide is deposited onto the frontside of the device wafer as a protection layer, while another layer of silicon oxide is deposited and then patterned as a hard mask on the backside of the device wafer. The device wafer is then temporarily bonded to a 6-inch, 675-*μ*m-thick Si handle wafer using the adhesive polymer (Santovac vacuum fluid), with the backside of the device wafer facing outward for backside etching. The DRIE process is conducted using the STS2 Inductively Coupled Plasma (ICP) etch system, with alternating cycles of etching and protective polymer deposition to achieve a high aspect ratio. Specifically, octafluorocyclobutane (C_4_F_8_) is used as the passivation gas, while sulfur hexafluoride (SF_6_) and oxygen (O_2_) are used as the etchant gases. The BOX layer works as the etching stop layer due to the high etching selectivity between silicon and silicon oxide. Finally, the device wafer is debonded from the handle wafer through a 2-min treatment on a 90 °C hotplate, and dies of various designs are separated. The residual Santovac vacuum fluid is cleaned sequentially with acetone, isopropyl alcohol (IPA), and deionized (DI) water. Any residual buried oxide, frontside oxide, and backside oxide are removed by the buffered hydrofluoric acid (HF) treatment to release the device.

### Characterization

**Fig. 2:**
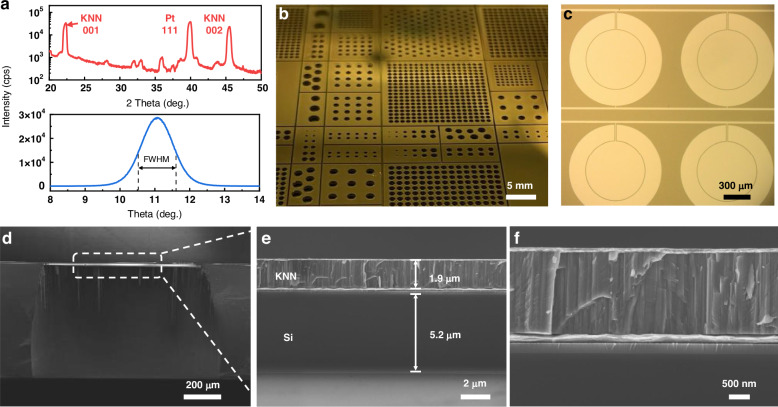
Characterization results. **a** X-ray diffraction (XRD) of the sputtered KNN film. The red line displays the locked couple scanning and the blue line is the 001 rocking curve. **b** Backside image of KNN pMUTs with varying sizes of etched holes. **c** A top-view microscope image of a prototype KNN pMUT array. Cross-sectional SEM images of: **d** a diaphragm with a backside cavity; **e** stacked diaphragm layers (Pt/RuO_2_/KNN/Pt/ZnO/SiO_2_/Si); and **f** the active piezoelectric layer with columnar structures

Figure 2 presents the characterization results of the fabricated KNN pMUTs. The crystal structure of the sputtered KNN film is examined by using X-ray diffraction (XRD), as shown in Fig. 2a. The locked couple scanning results show the 001 and 002 diffraction peaks of the KNN layer, as well as the 111 diffraction peak of the Pt electrode. The full-width at half-maximum (FWHM) measured from the rocking curve about the 001 diffraction condition is ~1.08°, which confirms the good crystallinity of the KNN film. Figure 2b shows the backside image of pMUT devices with various hole sizes, demonstrating the flexibility in design and fabrication for devices with different resonant frequencies. The optical top-view image of pMUTs (Fig. 2c) highlights the benign surface morphology. The cross-sectional scanning electron microscope (SEM) images in Fig. 2d–f display the well-defined backside silicon cavity, the tightly stacked Pt/RuO_2_/KNN/Pt/ZnO/SiO_2_/Si multi-layered diaphragm structure, and the thickness of each layer (1.9-μm-thick KNN and 5.2-μm-thick Si device layer). Figure 2f presents a zoomed-in image of the active layer, where the dense columnar structure of the KNN film validates good crystal orientation for the high-quality films. Additionally, due to imperfections in the backside etching process, deviations exist between the actual and designed diaphragm radius (500 μm), as illustrated in Fig. 2d. For simulation and theoretical analysis in the next section, a diaphragm radius of 420 μm is used as an example.

### Electrical characterization

**Fig. 3:**
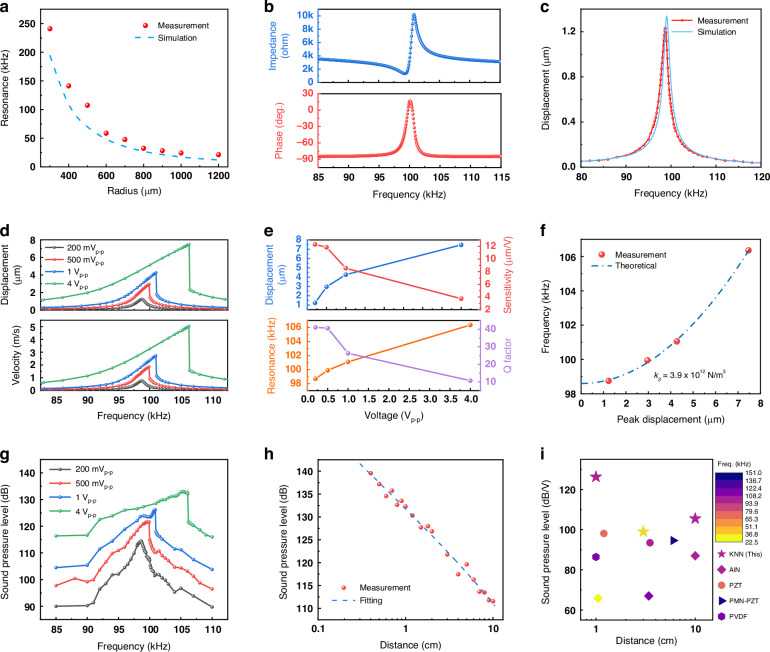
Electrical, mechanical, and acoustic characterizations. **a** Simulated (blue) and measured (red) resonant frequencies for different elements. **b** Measured impedance and phase versus frequency for a 500-μm-radius pMUT driven by a 200 mV_p-p_ voltage at the inner electrode. **c** Simulated (blue) and measured (red) center displacement of a pMUT (500 μm in radius) under a 200 mV_p-p_ differential driving. **d** Measured center displacement and velocity versus frequency under various excitation voltages (200 mV_p-p_, 500 mV_p-p_, 1 V_p-p_, 4 V_p-p_) with the differential driving scheme. **e** Resonant frequency, center displacement, displacement sensitivity, and quality factor versus driving voltage. **f** Measured (red) and fitted (blue, to Eq. (3)) resonant frequency with respect to peak displacement. **g** Measured root mean square (RMS) sound pressure level versus frequency under the differential driving scheme at a 1 cm axial distance. **h** Measured (red) and fitted (blue, to Eq. (4)) RMS sound pressure level versus axial propagation distance at 106.3 kHz under 4 V_p-p_. **i** Transmission performance for state-of-the-art pMUTs

The electrical impedance characteristics of single KNN pMUT elements are measured by an impedance analyzer under 2 V_p-p_. As the diaphragm radius increases from 300 to 1200 μm, the resonant frequency decreases from 241 to 21.2 kHz in Fig. 3a. This decrease is attributed to the inverse relationship between the resonant frequency of the diaphragm under flexural mode and the square of the diaphragm radius^47^. The deviations between the simulation and experimental results are attributed to differences in the actual diaphragm radius, as well as the residual stress within the films. Notably, the simulated results for a radius of 400–420 μm closely match the measured resonant frequency with the designed radius of 500 μm. The impedance and phase versus frequency curves of a pMUT element (500 μm in radius) obtained under a 200 mV_p-p_ excitation across its inner electrode and bottom electrode is shown in Fig. 3b. A resonant frequency ${f}_{r}$ of 99.29 kHz and an anti-resonant frequency ${f}_{a}$ of 100.79 kHz are observed. The electromechanical coupling factor ${k}_{t}^{2}$ is calculated to be 3.62% based on the following equation^35^:

1 $${k}_{t}^{2}=\frac{{\pi }^{2}}{4}\frac{{f}_{r}}{{f}_{a}}\frac{{f}_{a}-{f}_{r}}{{f}_{a}}$$

As such, the electromechanical coupling factor of the unimorph, clamped KNN pMUT is ~2.5 times higher than that of AlN-based pMUTs (~1%) and about 1.8 times that of ScAlN-based pMUTs (~2%)^34,35^.

### Mechanical characterization

The mechanical vibration behavior of the KNN pMUT (500 μm in radius) under the differential driving scheme is characterized using a single-beam laser Doppler vibrometer (LDV, Polytec VibroOne) with continuous sine-wave excitation. Detailed information is available in the “Experimental setup” section. Figure 3c displays both the simulated and measured frequency response of the displacement amplitude at the center of the diaphragm under a 200 mV_p-p_ excitation. This center position also corresponds to the point of maximum displacement across the diaphragm in the fundamental mode shape. Model details are described in the “Simulation method” section. A symmetrical frequency response is observed in both cases, and the measured displacement reaches 1.23 μm under a driving voltage of only 200 mV_p-p_, corresponding to a displacement sensitivity of 12.3 μm per volt (μm/V). The deviations between the simulation and experimental results are attributed to differences in the actual diaphragm radius, residual stress within the films, mechanical and piezoelectric material properties. Figure 3d further illustrates the mechanical performance under various excitation voltages, including 200 mV_p-p_, 500 mV_p-p_, 1 V_p-p_, and 4 V_p-p_. Both displacement and velocity increase with voltage. The center displacement at resonance under 4 V_p-p_ can reach 7.48 μm, showing a high displacement sensitivity of 3.74 μm/V, which is 3.3 times higher than that of a 36% scandium-substituted AlN pMUT (0.86 μm/V at 85 kHz)^33^. This result also matches well with the theoretical predictions based on the piezoelectric coefficient ${e}_{31,f}$. The frequency response gradually becomes asymmetrical as the voltage increases, where the nonlinear hardening behavior can be observed starting from 500 mV_p-p_, which is caused by the membrane tensioning effect^49^.

The impacts of driving voltage on resonant frequency, center displacement at resonance, displacement sensitivity, and quality factor are summarized in Fig. 3e. With increased driving voltage, the resonance shifts from 98.7 to 106.3 kHz. The corresponding displacement increases from 1.23 to 7.48 μm, while displacement sensitivity is reduced from 12.3 to 3.74 μm/V, indicating the reduced driving efficiency in the nonlinear region. The quality factor (Q) also decreases from around 41 to around 11 correspondingly, indicating a larger damping and faster mechanical-acoustic energy transfer process.

We further investigate the nonlinear behavior of the pMUT, which is found to be dominated by Duffing nonlinearity (${k}_{3}{x}^{3}$). Therefore, the governing equation of motion can be described as^50^:

2 $$\ddot{x}+2\left({\zeta }_{1}+{\zeta }_{3}{x}^{2}\right){\omega }_{0}\dot{x}+{\omega }_{0}^{2}x+\frac{{k}_{3}}{{m}_{{eff}}}{x}^{3}=\frac{{F}_{{ext}}}{{m}_{{eff}}}$$

where ${\omega }_{0}=2\pi {f}_{0}$ is the angular eigenfrequency of the fundamental flexural mode; ${m}_{{eff}}$ is the effective modal mass of the device; ${F}_{{ext}}$ is the external driving force on the pMUT; ${k}_{3}[N/{m}^{3}]$ is the coefficient of the conservative Duffing nonlinearity; ${\zeta }_{1}$ is the linear damping coefficient; and ${\zeta }_{3}[{m}^{-2}]$ is the third order nonlinear damping coefficient. The nonlinear stiffness term involving ${k}_{3}$ induces amplitude-dependent frequency drift, as observed in Fig. 3d. Meanwhile, the nonlinear damping term involving ${\zeta }_{3}$ creates amplitude-dependent decay, which results in the Q variation. By solving the differential equation, we can deduce the steady-state resonant frequency under increasing displacement $r$:

3 $$\omega ={\omega }_{0}+\frac{3{k}_{3}{r}^{2}}{8{\omega }_{0}{m}_{{eff}}}$$

Figure 3f depicts the nonlinear behavior of the pMUT in the resonant frequency versus the peak displacement plot. Equation (3) is used to fit the measurement results. In the equation, the angular eigenfrequency ${\omega }_{0}=2\pi {f}_{0}$ is derived using the eigenfrequency (${f}_{0}=98.7{kHz}$) extracted from Fig. 3d when the pMUT is operating within the linear region. The effective modal mass (${m}_{{eff}}=2.71\times {10}^{-9}{kg}$) is calculated as ${m}_{{eff}}=0.184\pi {a}^{2}\sum _{i=1}{{\rho }_{i}h}_{i}$, where $a$ is the radius of the pMUT, and ${\rho }_{i}$, ${h}_{i}$ denote the density and thickness of the ${i}_{{th}}$ layer respectively (cross-section shown in Fig. 1a). The coefficient 0.184 is derived from the lumped parameter when equating the flexural mode pMUT to a piston vibrator^47^. Subsequently, we fix the ${\omega }_{0}$ and ${m}_{{eff}}$ in the equation to fit the observed nonlinear behavior in Fig. 3f and the Duffing coefficient ${k}_{3}$ is extracted from the optimal fitting result as $3.9\times {10}^{12}N/{m}^{3}$. This coefficient primarily arises from geometrical nonlinearity, which can be expressed as ${k}_{3}=\frac{6.24\pi h{E}_{Y}}{{d}^{2}}\frac{13+21\nu -4{\nu }^{2}}{30(1+\nu )}$, where $h$, $d$, ${E}_{Y}$, and $\nu$ are thickness, diameter, Young’s modulus, and Poisson’s ratio of the diaphragm materials^51^, respectively.

### Transmission performance

The transmission pressure of the prototype KNN pMUT (500 μm in radius) under the continuous differential sine-wave driving is evaluated using a high-sensitivity microphone (Bruel & Kjar, Type 4138). The receiving signal is collected by placing the microphone 1 cm above the pMUT, and the root mean square (RMS) sound pressure level (SPL) is extracted, as shown in Fig. 3g. Detailed setup and calculations are provided in the “Experimental setup” section. The transmission pressure behaviors are similar to those of mechanical characteristics. As the transmission pressure increases with a high driving voltage, the resonant frequency also increases. A maximum transmission pressure of 133 dB RMS SPL is achieved at 105.4 kHz with a 4 V_p-p_ drive voltage. The effect of axial propagation distance on transmission pressure is further evaluated at 4 V_p-p_, as shown in Fig. 3h. The transmission pressure gradually decreases from 139.5 dB at 0.4 cm to 132.3 dB at 1 cm, and 111.6 dB at 10 cm (at the resonance of 106.3 kHz during testing). Thus, the transmission sensitivity of the single KNN pMUT reaches 126.3 dB/V RMS SPL at 1 cm and 105.5 dB/V RMS SPL at 10 cm axial distance. The output pressure decays exponentially as the distance $R$ increases, and this matches well with the theoretical model^52^:

4 $$p\left(R\right)=\frac{\rho {f}_{0}{S}_{T}{V}_{T}}{R}{10}^{-\alpha R}$$

where $\rho$ is the medium density; ${S}_{T}$ is the output volume velocity sensitivity of pMUT; ${V}_{T}$ is the transmit voltage; and $\alpha$ is the attenuation coefficient under operating frequency ${f}_{0}$.

A summary of the transmission performance comparison with other devices in the state-of-the-art literature is presented in Fig. 3i and Table 1. The sound pressure level correlates with the operation frequency (resonant frequency). A high operation frequency can yield a high output pressure but also experiences large attenuation (large attenuation coefficient $\alpha$) during propagation. These characteristics make it challenging to evaluate pMUT performance across a broad range of frequencies. In Fig. 3i, the x-axis is the distance, and the y-axis is the pressure output. The frequency information is color-coded for devices from 20 to 150 kHz. Different piezoelectric materials are indicated with various markers. From the results, the KNN pMUT stands out with its transmission outputs 5 to 10 times higher than those of AlN pMUTs, and better than those of PZT-based pMUTs reported in these literature. This highlights its exceptional advantage of achieving high SPL under a low-driving voltage.

**Table 1:** Transmission sensitivity comparison with other devices in the state-of-the-art literature

Material	Frequency	V_p-p_	SPL (dB)	SPL (dB/V)	Distance (cm)	Year	Ref.
AlN	101k	20	110	90	10	2021	^14^
AlN	35.3k	32	89.9	65.8	1.05	2020	^76^
PZT	100k	6	103	93.5	3.5	2017	^37^
PZT	482k	2	63.7	63.7	1	2016	^77^
PMN-PZT	151k	3	98.1	94.6	6	2017	^78^
PVDF	121.5k	10	100.4	86.4	1	2018	^19^
**KNN**	**106.3k**	**4**	**132.3**	**126.3**	**1**	**This work**	
**KNN**	**106.3k**	**4**	**111.6**	**105.5**	**10**	**This work**	
**KNN**	**22.8k**	**4**	**105**	**99**	**3**	**This work**	

### Mid-air haptic application

Haptic interfaces can enrich human-machine interactions by providing tactile feedback via the stimulation of mechanoreceptors located beneath the skin. Current haptic actuators, including the eccentric rotating mass (ERM) and linear resonant actuator (LRA), utilize electromagnetic force to produce low-frequency vibrations (<1 kHz). These actuators typically face limitations on operating within a narrow frequency range and exhibit significant actuation time lag^53^. Recently, mid-air haptic stimulation has gained interest for its advantages in fast actuation, fine spatiotemporal resolution^54^, and immunity from skin surface variations. Touchless haptic systems based on bulk ultrasonic transducers, however, have a very large form factor (tens of centimeters in width), which is unsuitable for hand-held or wearable applications. Haptic actuators based on AlN pMUTs^17,18^, PVDF-based pMUTs^19,55^, and LiNbO_3_ pMUTs^56^ have been reported. For example, a 12 × 12 AlN pMUT array^17^ with a resonant frequency of 109.4 kHz can generate a pressure amplitude of 950 Pa at 15 mm away under a 20 V_p-p_ excitation. By focusing the acoustic waves, a polymeric P(VDF-TrFE) annular pMUT array^55^, resonating at 390 kHz, can generate a focal pressure of 1600 Pa at 20 mm away under a 63 V_p-p_ excitation. However, the acoustic output pressure of these devices is limited due to the intrinsically small piezoelectric coefficient ${e}_{31,f}$ of active materials, resulting in a vague tactile sensation.

**Fig. 4:**
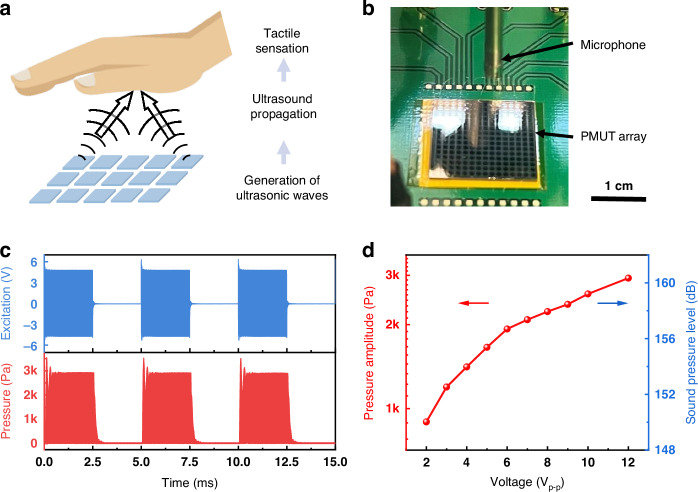
Performance evaluation of KNN pMUT array as a haptic actuator. **a** The haptic stimulation on the human palm by inducing mechanical vibrations on mechanoreceptors in human tissues. **b** Pressure measurement setup using a microphone in ambient air. **c** The excitation signal (12 V_p-p_, blue) and the output pressure (red) measured at the natural focal point of the pMUT array, 15 mm away from the array surface. **d** Output pressure amplitude and RMS sound pressure level versus applied voltage under the differential driving scheme

Here, we present a mid-air haptic interface device enabled by an array of 15 × 15 KNN pMUT elements^57^. As illustrated in Fig. 4a, the ultrasonic waves generated by the pMUT array propagate through the air and induce vibrations on the mechanoreceptors upon reaching the air-skin interface, thereby generating tactile sensations. The prototype pMUT array covers a total area of 2 cm by 2 cm, and the pressure field evaluation reveals that the natural focal point of the array without beam focusing is ~15 mm from the diaphragm surface, as shown in Fig. 4b. The output pressure evaluations are also conducted in the focal area to maximize performance. The transmitted ultrasound is modulated to low-frequency signals via the pulse-width modulation (PWM) scheme, as the ultrasound frequency signals are not perceptible to mechanoreceptors. As shown in Fig. 4c, the excitation signal features a 92.4 kHz carrier frequency, which corresponds to the resonant frequency of the pMUT array. This signal is modulated to 200 Hz, which lies within the more sensitive frequency range of mechanoreceptors^58^. Both carrier and modulation signals are rectangular waves. The modulation signal features a 50% duty cycle from prior research for optimal sensation results^17,59^, which corresponds to a pulse width of 2.5 ms within a 5-ms period. The transmission performance is analyzed under various voltages, with results summarized in Fig. 4d. The focal pressure amplitude increases from 896 Pa at 2 V_p-p_ to 2900 Pa at 12 V_p-p_, which corresponds to an RMS sound pressure level of around 160.3 dB SPL. This represents the highest output pressure achieved using an airborne pMUT array as a mid-air haptic actuator. Additionally, with a driving voltage of only 12 V_p-p_, the transmitting sensitivity of 120.8 Pa/cm^2^/V is at least two times greater than that of AlN pMUTs at a similar frequency^17^. Since the achieved pressure exceeds the minimum sensing threshold of 1 kPa^18^, instant non-contact haptic stimulation, producing a wind-like sensation on human palms, has been realized in 90% of the volunteer tests^57^. The transmission outputs of the array can be further enhanced by increasing process uniformity across the wafer. Additionally, beamforming strategies can be utilized to focus the acoustic waves and improve the efficiency in acoustic energy utilization. Beamforming can be implemented by optimizing the array design and employing a multi-channel control scheme for customized excitation. Moreover, the focused beam can be steered in one or two dimensions through amplitude and phase engineering. This capability would enable more diverse haptic stimulation patterns.

### Loudspeaker application

**Fig. 5:**
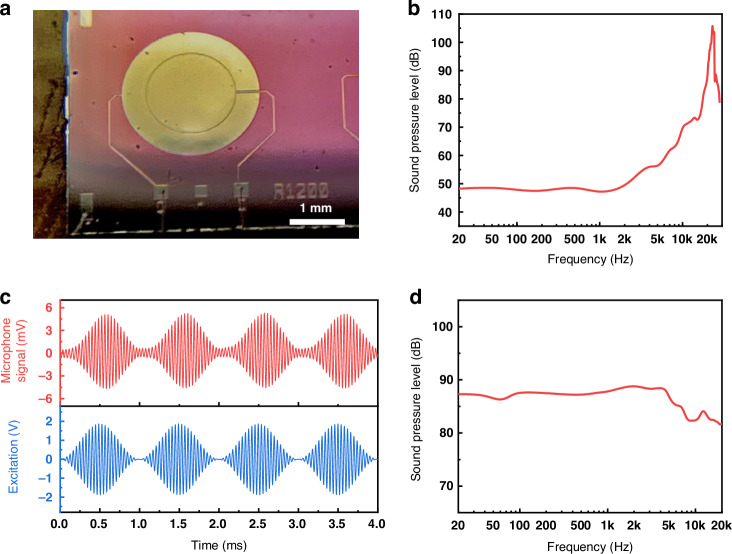
Performance evaluation of a single KNN pMUT as a loudspeaker. **a** The optical image of a single pMUT (1200 μm in radius) on a PCB board for the loudspeaker demonstration. **b** Measured sound pressure level covering the audible sound range (20 Hz to 20 kHz) at a 3 cm axial distance. **c** Amplitude modulation (AM) signals to drive the pMUT (1000 Hz modulation, blue) and measured output signals from a microphone (red). **d** Output SPL after modulation with a 24.8 kHz carrier frequency

MEMS loudspeakers have attracted extensive interest due to their small form factors, low power consumption, and ease of integration with analog front ends^60^. Unlike conventional loudspeakers, which rely on large components such as coils and magnets, MEMS loudspeakers can be realized by microfabrication processes with piezoelectric thin films as the active components. Here, we demonstrate the potential of KNN pMUTs as loudspeakers through a single pMUT element. A single pMUT with a resonant frequency close to the audible sound range is designed with a diaphragm radius of 1200 μm (Fig. 5a). Figure 5b shows the experimental frequency responses under a 4 V_p-p_ excitation, where a high SPL of 105 dB can be achieved near the resonant frequency of 22.8 kHz at a 3 cm axial distance. The SPL remains above 46 dB throughout the audible frequency range from 20 Hz to 20 kHz. With proper structure optimization^61^ and acoustic packaging^62^, a more uniform frequency response could be achieved.

An alternative way to achieve audible sound through ultrasonic transducers is signal modulation and utilizing the nonlinear acoustic effect^63^, such as amplitude modulation^64^, which is also a way to realize directional loudspeakers. Figure 5c shows the amplitude modulation scheme commonly used in parametric arrays^65^. For better reliability and to avoid buckling, we choose to operate this pMUT at 24.8 kHz under a 4 V_p-p_ excitation, which is slightly higher than its resonant frequency. By modulating this high-frequency carrier wave (${f}_{c}=24.8{kHz}$) with the audible-frequency modulating wave ${f}_{m}$, the dual-band excitation signal can be constituted as^66^:

5 $$\begin{array}{l}{y}_{m}\left(t\right)=A\sin \left(2\pi {f}_{c}t\right)+\frac{1}{2}{Am}\left[\sin \left(2\pi \left({f}_{c}+{f}_{m}\right)t+\phi \right)\right.\\\qquad\quad\;\;+\,\left.\sin \left(2\pi \left({f}_{c}-{f}_{m}\right)t-\phi \right)\right]\end{array}$$

where $A$ and $m$ are the amplitude of the carrier wave and modulation wave, respectively, and $\phi$ is the phase of the modulating signal. The ${f}_{c}+{f}_{m}$, ${f}_{c}$ and ${f}_{c}-{f}_{m}$ components will interact with each other through the nonlinear effect of the air^67^ and generate low-frequency audible sound. This audible sound can be confirmed through the envelope of the output pressure measured at 3 cm axial distance after digital filtering. Furthermore, we take the amplitude of the envelope signal to evaluate the generated audible sound. The frequency response of the modulated signal is illustrated in Fig. 5d, indicating that the single KNN pMUT can generate a uniform acoustic output with an SPL of around 85 dB (with a deviation of ±3 dB) when modulated within the audible frequency band of 20 Hz–20 kHz. In brief, these results show that the KNN pMUT can effectively generate acoustic signals in the audible sound range. Although this structure is not optimized for speaker applications, the successful generation of audible sound demonstrates the strong output of the KNN pMUT. With further structure designs, such as suspension-spring design^61^, or cantilever plate design^68^ and package optimizations, such as back-chamber design^69^, the KNN pMUT has the potential to exhibit high SPL and a flat frequency response.

### Airborne rangefinder application

Apart from functioning as acoustic transmitters, ultrasonic transducers can sense incident ultrasounds. Ultrasound Time-of-Flight (ToF) sensors record the propagation time of a transmitted signal as it reaches a target and reflects back. This process is not affected by light conditions or the colors of targets, making them ideal for object detection, distance measurements, and positioning^70^. Recently, air-coupled pMUTs have shown great potential as ultrasonic rangefinders in consumer electronics, such as low-power presence sensing^71^ and AR/VR applications^10^. The detection range depends on the transducer’s transceiving sensitivity, the noise floor of the readout electronics, and the acoustic path loss during propagation^72^. The transceiving sensitivity of a ToF sensor is proportional to $\frac{{{e}_{31,f}}^{2}}{{\varepsilon}_{r}}$. AlN-based rangefinders are limited in transmission due to their small piezoelectric coefficient ${e}_{31,f}$, whereas PZT-based rangefinders suffer from low receiving performance due to their high dielectric constant ${\varepsilon }_{r}$. In contrast, the high ${e}_{31,f}$ and relatively low ${\varepsilon }_{r}$ of KNN make it promising to extend the operation distance. Here, we characterize the performance of KNN pMUTs as an airborne rangefinder via pulse-echo measurements with a single KNN pMUT. The outer electrode serves as the transmitter, and the inner electrode works as the receiver to ensure a resonant frequency match. As previously discussed, a high-frequency ultrasound wave typically yields a high output pressure, but experiences greater acoustic path loss due to rapid attenuation during propagation. Additionally, higher frequencies can provide better temporal and lateral resolutions due to their shorter wavelengths. In this demonstration, we use a KNN pMUT element with a radius of 500 *μ*m that was characterized in earlier sections. For practical applications, the operation frequency depends on a proper balance among output pressure, attenuation rate, and detection resolution.

**Fig. 6:**
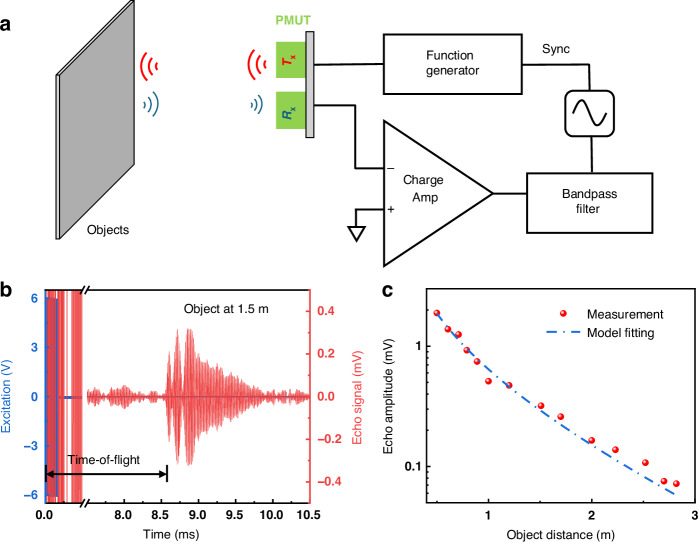
Performance evaluation of a single KNN pMUT as an airborne rangefinder. **a** Schematic of the pulse-echo measurement setup. **b** Excitation signals (blue) for the transmitting electrode and collected echo signals (red) at the receiving electrode with an object about 1.5 m away. **c** Echo amplitudes at various object distances. The blue line is a fit to Eq. (4), where the travel distance, R, is twice the object distance, and an additional term is used to represent the target reflectivity in the equation

The setup for pulse-echo measurement is shown in Fig. 6a. A plastic board is used as an object to reflect pulsed ultrasound signals emitted by the pMUT transmitter, and the corresponding echo signals from the receiver are collected by an oscilloscope after being processed by a charge amplifier and bandpass filter. During the measurement, the transmitting electrode is driven by a 19-cycle pulsed square wave (102.7 kHz, 12 V_p-p_), and the receiver signal is constantly monitored. To determine the detection range, the object distance is extended until the echo signal merges with the noise floor and the undesired reflections from the surrounding environment. An example echo signal is shown in Fig. 6b when the object is 1.5 m away. The echo has a magnitude of 0.32 mV and a ToF around 8.6 ms, which is calculated so that the received echo signal is at least 2 times larger than the baseline signal. The pMUT can detect up to a distance of 2.82 m (Fig. 6c), demonstrating its good pulse-echo sensing ability. The echo amplitude decay also matches the model in Eq. (4). The travel distance $R$ is twice the object distance, and an additional target reflectivity term^52^ should be added. To further extend the sensing range, we can design an array of pMUT elements^33,73^ to improve transmission outputs and widen the field of view. The readout electronics can be customized to enhance the SNR^52^. Developing suitable acoustic packages^72,74^, such as exponential horn^72^ and tube resonator^74^, is another way to further improve the range by enhancing the acoustic coupling with the medium.

## Conclusions

This work demonstrates sputtered KNN-based pMUTs, with the KNN film exhibiting good crystal quality in the 001 orientation for the high piezoelectric coefficient. A single KNN pMUT with a resonant frequency of 106.3 kHz under 4 V_p-p_ is used to evaluate the transmission performances and obtains a large vibration amplitude of up to 3.74 μm/V and velocity of 2.52 m/s/V. Benefiting from the high surface velocity, a transmission pressure of 132.3 dB SPL can be obtained at an axial distance of 1 cm and 111.6 dB at an axial distance of 10 cm. The corresponding transmission sensitivity is 5–10 times higher than those of state-of-the-art AlN-based pMUTs. Further research could include structural design to enhance the multi-domain coupling effects with expanded bandwidth, packaging designs to improve acoustic coupling with the environment, customized electronics to improve driving efficiency, and environmental sensitivity and long-term reliability study for practical applications.

Potential applications such as haptic actuators, loudspeakers, and rangefinders have been demonstrated and discussed. For the haptic application, a 15 × 15 KNN pMUT array achieves a focal pressure amplitude of 2900 Pa. This could be further enhanced by optimizing the array design or applying beamforming techniques. For the loudspeaker application, a single pMUT element with a resonant frequency near the audible range demonstrates stable acoustic output by the amplitude modulation scheme. For the airborne rangefinder application, the pulse-echo measurements of a single pMUT element exhibit excellent sensing capabilities, maintaining a distinguishable echo signal at distances up to 2.82 m. As such, this work sheds light on high-SPL and low-driving-voltage pMUTs for potential applications in various fields where high acoustic pressure and compact form factors are favorable. Potential applications include acoustic cooling, portable and wearable ultrasound imaging, cardiovascular and edema monitoring, intravascular ultrasound (IVUS), external and interstitial high-intensity focused ultrasound (HIFU), non-destructive testing, flowmeter, underwater imaging, particle manipulation, and acoustic tweezers.

## Materials and methods

### Simulation method

Finite element analysis (FEA) is used to create a simulation model in COMSOL Multiphysics® v. 5.6 with three domains: Solid Mechanics to simulate the deformation of the diaphragm (fixed boundary condition), Electrostatics to simulate the internal electric field across the piezoelectric material, and Pressure Acoustics to simulate the propagation of sound waves. The Piezoelectric Effect and the Acoustic-Structure Boundary multiphysics components are applied to generate sound waves by the diaphragm. A 2D axisymmetric model is adopted to reduce computational complexity, as demonstrated in Fig. S1, due to the geometric symmetry. Free triangular meshes are used with a maximum size of one-tenth of the sound wave wavelength. A 420-μm-radius diaphragm is used as the model with a 2-μm-thick KNN layer and a 5-μm-thick silicon structural layer. Young’s modulus, density, and Poisson’s ratio of the KNN layer are set to 65 GPa, 4000 kg/m^3^, and 0.32, respectively, with the piezoelectric coefficient ${e}_{31,f}$ at −8 C/m^2^. The silicon structural layer is set to have a Young’s modulus of 169 GPa, a density of 2320 kg/m^3^, and a Poisson’s ratio of 0.22. A voltage of 200 mV_p-p_ is applied on the two regions of the top electrode by using the differential drive mechanism with the bottom electrode grounded to 0 V. The acoustic domain has a hemispherical air region with a radius of 50 mm, with the infinite far-field set at a radius of 40 mm centered on the KNN layer. Frequency domain analysis is conducted between 80 and 120 kHz to solve for the resonant frequencies and the displacement of the diaphragm.

### Experimental setup

During the experiments, the pMUT chips are affixed to a printed circuit board (PCB) using tapes, and the electrodes are connected to the gold (Au) contacts on the PCB via an aluminum (Al) wire-bonding process. There are two driving schemes. When the differential driving scheme is used, the bottom electrodes are grounded, and the excitation signals applied to the inner and outer top electrodes have a 180° phase shift. When only the inner electrode is used, the outer electrode is left unconnected. To evaluate the mechanical characteristics, a single-beam laser Doppler vibrometer (LDV, Polytec VibroOne) is used to capture the displacement and velocity information^75^, which is then recorded by a data acquisition system (PicoScope, 5000 series) with the experimental platform shown in Fig. S2a. The laser point is focused on the center of the diaphragm in Fig. S2b, and the measurement chamber is isolated from the ambient environment to minimize interferences. The raw data captured from the LDV is presented in the format of voltage, corresponding to the preset velocity sensitivity (options used: 500 mm/s/V or 5 m/s/V) and displacement sensitivity (options used: 500 nm/V, 5 μm/V, or 50 μm/V). Both sensitivities are adjusted accordingly based on the amplitude of the captured data. These raw data are subsequently converted into displacement and velocity information during post-signal processing. The quality factor (Q) is calculated based on the -6 dB bandwidth from the displacement frequency response. To characterize the acoustic performance, a microphone (Bruel & Kjar, Type 4138) equipped with a preamplifier (Type 1708) is used to measure the output pressure, as shown in Fig. S3. The raw microphone signal is recorded by the data acquisition system as voltages and then converted to pressure (Pa) using the microphone sensitivity (0.719 mV/Pa) and free-field calibrations. The sound pressure level is calculated from the root mean square (RMS) pressure amplitude based on the following equation:

$${{\mathrm{SPL}}}=20{\log }_{10}\left(\frac{P}{{P}_{0}}\right)$$

Where $P$ is the measured sound pressure amplitude, and ${P}_{0}$ is the reference sound pressure, which is 20 μPa in air.

## Supplementary information

Supplementary Information
