# Supplementary material for: High sound pressure piezoelectric micromachined ultrasonic transducers using sputtered potassium sodium niobate
Source: Microsyst Nanoeng. 2024 Dec 27;10:205. doi: 10.1038/s41378-024-00841-y (PMC11671594; doi:10.1038/s41378-024-00841-y)
Supplement: Supplementary file 1 — Supplementary Information [file 41378_2024_841_MOESM1_ESM.docx]

**
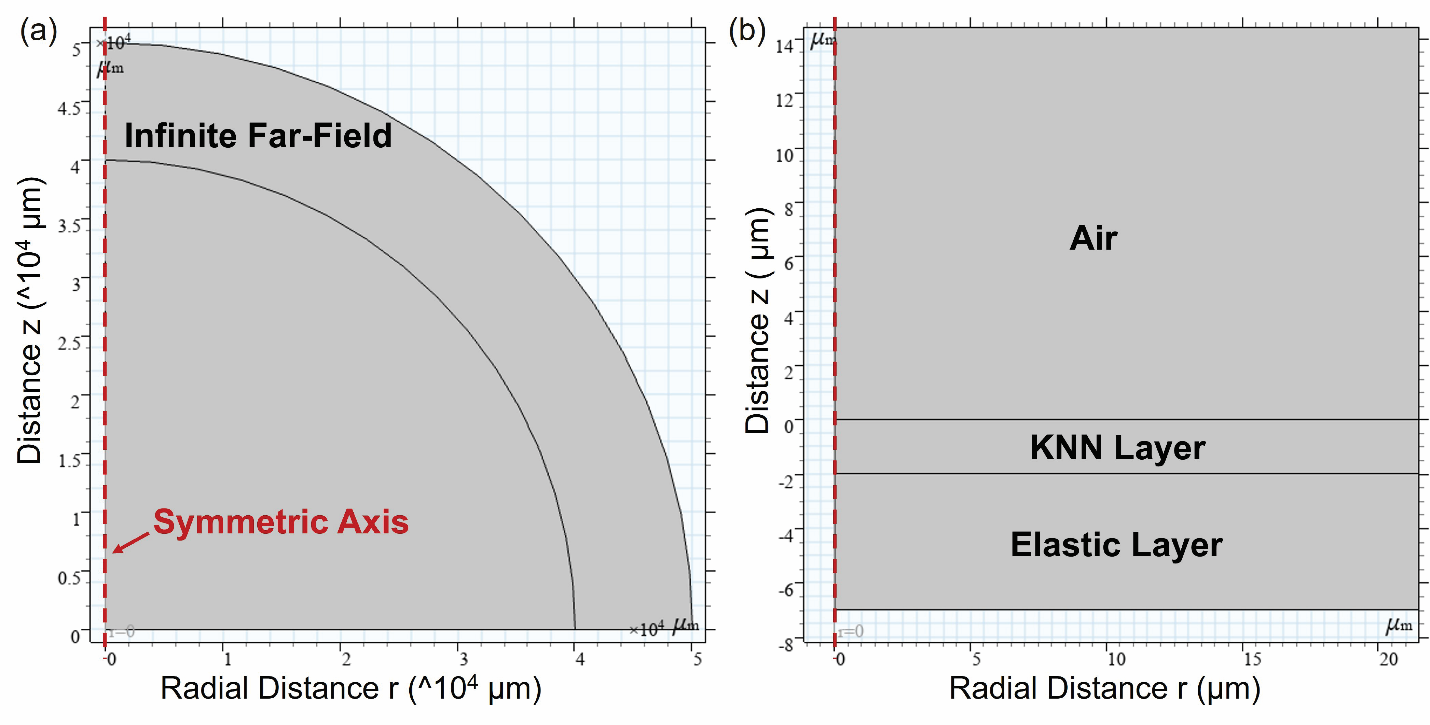
**

**Figure S1 | Geometric information of the simulation model.** (a) Air domain with the infinite far-field at 40 mm. (b) The fixed-boundary diaphragm has a radius of 420 $\mu m$ with a KNN layer, a silicon elastic layer, and an air region.


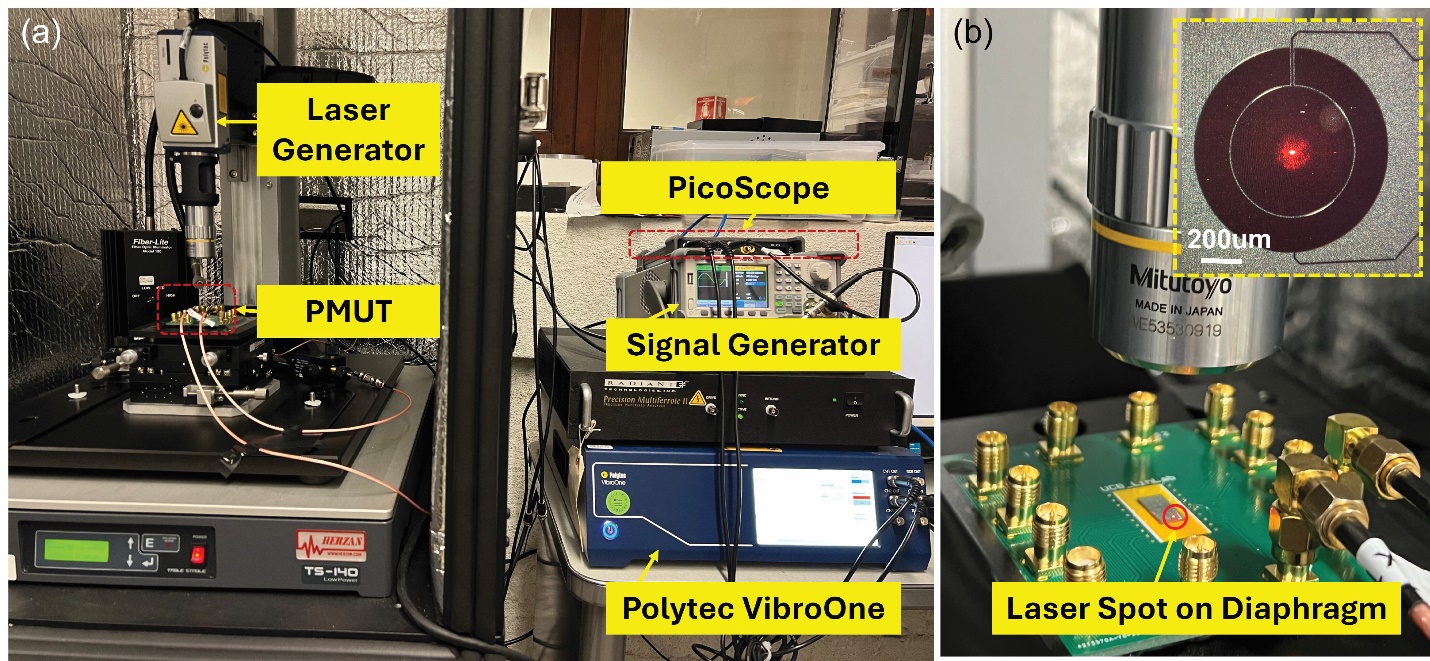


**Figure S2 | Experimental platform for displacement and velocity measurement.** (a) The setup includes a laser to emit light onto the diaphragm, a signal generator to excite the pMUT, a laser Doppler vibrometer (Polytec VibroOne) with its digital interface to measure the displacement and velocity at the laser spot area, and a PicoScope for data acquisition. (b) The laser point is focused on the center of the diaphragm.


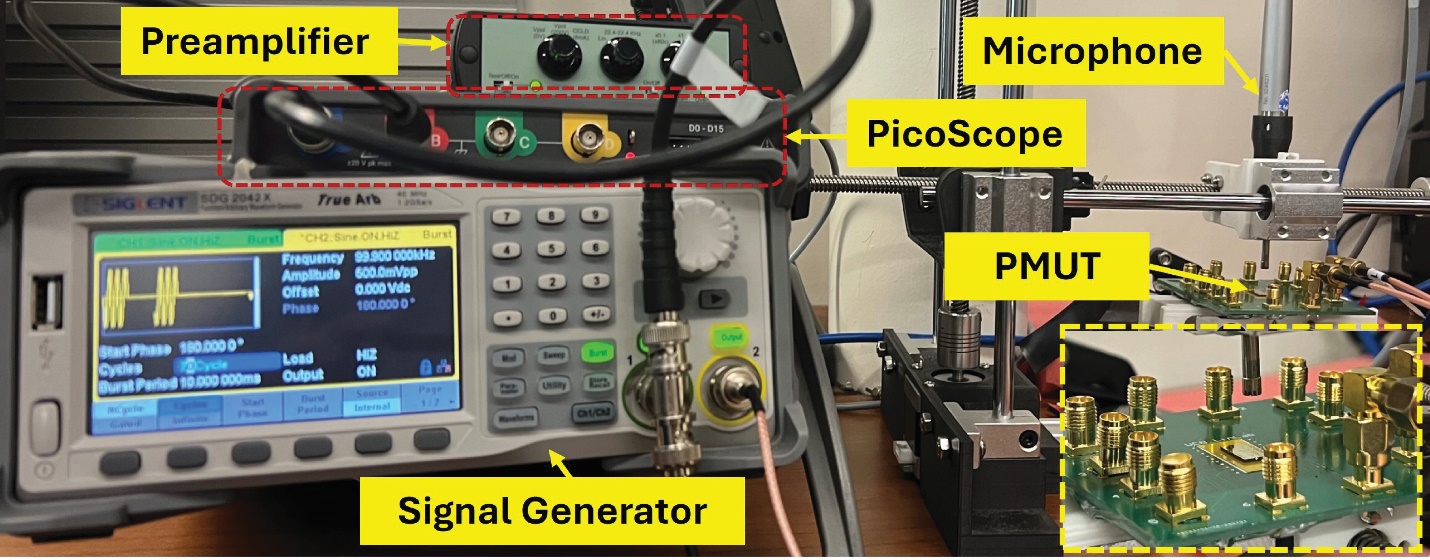


**Figure S3 | Experimental platform for sound pressure measurement.** The setup includes a signal generator to excite the pMUT, a microphone to detect output pressure, a preamplifier for signal conditioning and amplification, and a PicoScope for data acquisition.
